# 

**Published:** 1893-05

**Authors:** 


MAY, 1893.

### THE AMERICAN JOURNAL OF DENTAL SCIENCE.

EDITED BY

F. J. S. GORGAS, M. D., D. D. S.
RICHARD GRADY, M. D., D. D. S.

PUBLISHED MONTHLY.

$2.50 PER ANNUM.

Vol. 27. THIRD SERIES No. 1.

BALTIMORE:

SNOWDEN & COWMAN, 9 W. FAYETTE STREET.

LONDON:

TRUBNER & CO., 60 PATERNOSTER ROW

Entered at the Post Office at Baltimore, Md., as Second-class Matter.

PAYABLE IN ADVANCE.

